# Rat precision-cut liver slices predict drug-induced cholestatic injury

**DOI:** 10.1007/s00204-017-1960-7

**Published:** 2017-04-08

**Authors:** Viktoriia Starokozhko, Rick Greupink, Petra van de Broek, Nashwa Soliman, Samiksha Ghimire, Inge A. M. de Graaf, Geny M. M. Groothuis

**Affiliations:** 1 Division of Pharmacokinetics Toxicology and Targeting, Groningen Research Institute for Pharmacy, University of Groningen, Groningen, The Netherlands; 2 Department of Pharmacology and Toxicology, Radboud University Medical Center, Nijmegen, The Netherlands

**Keywords:** Drug-induced cholestasis, Precision-cut liver slices, Bile acids, Drug-induced liver injury

## Abstract

Drug-induced cholestasis (DIC) is one of the leading manifestations of drug-induced liver injury (DILI). As the underlying mechanisms for DIC are not fully known and specific and predictive biomarkers and pre-clinical models are lacking, the occurrence of DIC is often only reported when the drug has been approved for registration. Therefore, appropriate models that predict the cholestatic potential of drug candidates and/or provide insight into the mechanism of DIC are highly needed. We investigated the application of rat precision-cut liver slices (PCLS) to predict DIC, using several biomarkers of cholestasis: hepatocyte viability, intracellular accumulation of total as well as individual bile acids and changes in the expression of genes known to play a role in cholestasis. Rat PCLS exposed to the cholestatic drugs chlorpromazine, cyclosporine A and glibenclamide for 48 h in the presence of a 60 μM physiological bile acid (BA) mix reflected various changes associated with cholestasis, such as decrease in hepatocyte viability, accumulation and changes in the composition of BA and changes in the gene expression of *Fxr, Bsep* and *Ntcp*. The toxicity of the drugs was correlated with the accumulation of BA, and especially DCA and CDCA and their conjugates, but to a different extent for different drugs, indicating that BA toxicity is not the only cause for the toxicity of cholestatic drugs. Moreover, our study supports the use of several biomarkers to test drugs for DIC. In conclusion, our results indicate that PCLS may represent a physiological and valuable model to identify cholestatic drugs and provide insight into the mechanisms underlying DIC.

## Introduction

Drug-induced liver injury (DILI) is one of the main reasons of drugs being excluded during the drug-development process or withdrawn from the market (Yang et al. 2013). Therefore, many efforts have been made to select reliable biomarkers and develop predictive in vitro models to detect hepatotoxic effects as early as possible in the drug-development process.

Drug-induced cholestasis (DIC) is the leading and one of the most severe manifestations of DILI (Qiu et al. 2016). Cholestasis is characterized by a reduction in bile flow and can be caused by a variety of intra- or extra-hepatic mechanisms. Numerous drugs have been identified as a potentially cholestatic compound. And even though drugs undergo a screening for inducing cholestatic disorders in the pre-clinical and clinical phases, in many cases the occurrence of DIC is only being reported when the drug has been approved for registration and is administrated to thousands of patients. This is due to the fact that exact underlying mechanisms for DIC are not clear yet, hampering the validation of predictive biomarkers. At this moment there are no specific, highly predictive biomarkers for DIC (Padda et al. 2011). Elevations in the systemic blood levels of liver enzymes (such as alkaline phosphatase (ALP) and gamma-glutamyl transpeptidase (GGT)), bilirubin and bile acid (BA) are the most commonly used biomarkers in the clinic to identify cholestatic injury (Padda et al. 2011; Schadt et al. 2016; Yang et al. 2013). However, elevation in ALP and GGT are also observed during other liver diseases and their elevation during intra-hepatic cholestasis sometimes is minor. Also, an elevation in the circulating BA concentration is not always associated with DILI (Rodrigues et al. 2014), and, on the other hand, some drugs cause asymptomatic cholestasis with respect to BA, where elevation in the enzymes is the only symptom (Padda et al. 2011).

One of the most common causes of drug-induced cholestasis is the inhibition of the bile salt export pump (ABCB11 or BSEP) (Yang et al. 2013). BSEP is responsible for the excretion of the recirculated as well as newly synthesized BA from the hepatocytes into the bile canaliculi. Therefore, the inhibition of this transporter leads to the accumulation of BA in hepatocytes, which is believed to be one of the main causes of the hepatotoxicity observed during cholestasis.

Prediction of cholestatic side effects of drugs relies mainly on animal experiments using the same biomarkers as described for patients, with the same drawbacks. Moreover, these in vivo experiments do not provide any mechanistic insight, but do cause high animal discomfort. Thus, in vitro tests with high predictive value that also provide insight into the mechanism are highly needed. Some in vitro models have been developed to predict drug-induced cholestasis, based on hepatocyte sandwich monocultures (Chatterjee et al. 2014a, b; Oorts et al. 2016). However, the decrease in drug-metabolizing enzyme activities and the variation in the expression of drug transporters during culture, affects the predictivity and reliability of this model. Moreover, when cell viability is used as read-out parameter for DIC, it should be realized that various liver toxicity mechanisms require the presence of other cell types besides hepatocytes and, therefore, cannot be properly predicted by monocultures. It has been recognized that drug-induced cholestasis is a multifactorial process, where cholestasis might be a primary as well as a secondary event leading to hepatotoxicity (Rodrigues et al. 2014). These are so-called mixed cholestatic and hepatocellular injuries, where various liver cell types are involved (Padda et al. 2011; Schadt et al. 2016). Furthermore, bile acid homeostasis itself is a complex process that involves not only hepatocytes, but also other cell types, for example, cholangiocytes. Therefore, models that include various liver cell types would be of added value.

Precision-cut liver slices (PCLS) have been recognized as a reliable and predictive ex vivo model for many toxicological and pharmacological studies (de Graaf et al. 2010; Vickers and Fisher 2013). Advantages of this model include the presence of all liver cell types in their natural environment and physiological polarized expression of membrane transporters. Previous studies have shown that transcriptomics analysis of PCLS exposed to various cholestatic drugs successfully detected liver toxicity processes associated with cholestasis (Szalowska et al. 2013; Vatakuti et al. 2016). However, the direct measurement of total intracellular bile acid concentrations has not been successfully performed, even though it might be an important biomarker in predicting drug–bile acid interactions (Yang et al. 2013). Therefore, in the present study we aimed to investigate whether PCLS reflect pathological changes related to cholestasis and thus can be used to predict DIC. We evaluated several biomarkers of cholestasis such as hepatocyte viability, intracellular accumulation of bile acids and changes in the expression of genes known to play a role in cholestasis. For that, we exposed rat PCLS to different concentrations of three well-known cholestatic drugs: chlorpromazine (CP), cyclosporine A (CS), and glibenclamide (GB), in the presence of a physiological concentration of a bile acid mix mimicking the bile acid concentration and composition in the rat portal vein. The addition of BA to the medium is essential to allow BA to reach toxic intracellular levels upon BSEP inhibition, especially when BA-induced toxicity is an endpoint. Several recent studies on sandwich-cultured hepatocytes (SCH) showed that it was only possible to detect toxicity of cholestatic drugs, when BA was added to the culture medium (Chatterjee et al. 2014b; Ogimura et al. 2011). The relevance and predictivity of PCLS for DIC was shown by assessing the potential of the compounds to cause cholestasis *ex vivo* by disturbing the BA homeostasis and by demonstrating a correlation between BA accumulation and viability.

## Materials and methods

### Animals

Male Wistar rats (weight 250–300 g) were obtained from Charles River (Sulzfeld, Germany). Animals were housed in a temperature- and humidity-controlled room on a 12-h light/dark cycle with food and tap water *ad libitum* (Harlan Laboratories B.V., Horst, The Netherlands). The rats were allowed to acclimatize for at least 7 days before starting the experiments. All experiments were started between 9.00 and 10.00 am to avoid influence of diurnal rhythm. All experiments were approved by the Animal Ethical Committee of the University of Groningen.

### Excision of rat liver

The liver was excised under isofluorane/O_2_ anesthesia and placed into ice-cold University of Wisconsin (UW) organ preservation solution (DuPont Critical Care, Waukegab, IL) until use.

### Preparation and incubation of rat PCLS

PCLS were prepared as described previously by de Graaf et al. with minor modifications (de Graaf et al. 2010). In brief, PCLS of 5 mm in diameter, about 250 µm in thickness and approximately 5 mg wet weight were used in this study. To get rid of cell debris and restore the ATP levels after the slicing procedure, the slices were pre-incubated for 1 h at 37 °C in a 12-well plate filled with 1.3 ml of Williams’ medium E (WME) (Life Technology) saturated with 80%O_2_/5%CO_2_/15%N_2_ and supplemented with 25 mM glucose and 50 µg/ml gentamycin (Invitrogen), while gently shaking 90 times per minute. Afterwards, PCLS were transferred to another 12-well plate filled with 1.3 ml of medium with or without the 60 μM bile acid mix (composition of BA mix is given in Table 1) in combination with CP [18, 27 or 36 μM], CS [1, 3 or 5 μM], GB [120, 150 or 180 μM] or the solvent DMSO (concentration during incubation ≤0.5%) and incubated for 48 h. Non-toxic, low and medium toxic concentrations of drugs were chosen based on recently published results (Vatakuti et al. 2015) and results from pilot experiments, which showed very steep concentration–effect curves (data not shown). Medium was refreshed after 24 h of incubation with medium containing the same initial drug concentration.

**Table 1:** Composition of rat bile acid mix

Composition of bile acids	Final concentration in the incubation medium (μM)
Cholic acid (CA)	33.24
Chenodeoxycholic acid (CDCA)	1.62
Deoxycholic acid (DCA)	0.88
Lithocholic acid (LCA)	0.04
Glycocholic acid (GCA)	0.63
Taurocholic acid (TCA)	8.92
Ursodeoxycholic acid (UDCA)	0.55
Taurochenodeoxycholic acid (TCDCA)	1.55
Taurohyodeoxycholic acid (THDCA)	6.37
Hyodeoxycholic acid (HDCA)	5.33
Glycoursodeoxycholic acid (GUDCA)	0.11
Taurodeoxycholic acid (TDCA)	0.68

### ATP and protein content of PCLS

Viability of PCLS was determined after 48-h incubation by means of the ATP content as described previously using the ATP Bioluminescence Assay Kit CLS II (Roche, Mannheim, Germany) (de Graaf et al. 2010). Protein content of the hPCLS was measured using the Bio-Rad DC Protein Assay (Bio-Rad, Munich, Germany) using a bovine serum albumin calibration curve as described (Starokozhko et al. 2015).

### LDH leakage

LDH leakage from the slice to the medium was used as a marker of cell damage. In brief, medium was collected after 24- and 48-h incubation, and stored at 4 °C until analysis (maximum 1 week). The LDH assay was performed using the Cyto Tox-ONE Homogenous Membrane Integrity Assay kit (Promega, Madison, USA) according to the manufacturer’s instructions with slight modifications. In brief, 50 µL of medium sample was mixed with 50 µL of the substrate mix in a black 96-well plate. The plate was kept for 10 min in the dark and the stop reagent was added afterwards to the mixture. Fluorescence was read immediately at excitation 560 nm and emission 590 nm.

### Total bile acid assay (TBA)

It is expected that in PCLS a part of the total BA pool resides in bile canaliculi. Therefore, to measure the intracellular concentration of bile acids in the slices, an extra washing step to empty the bile canaliculi was performed before collecting PCLS for the TBA analysis. After 48 h of incubation, the PCLS were transferred to another 12-well plate containing 1.3 ml of Ca^2+^-free and Mg^2+^-free Hank’s Balanced Salt Solution (HBSS) (Life Technology) saturated with 90% O_2_/5% CO_2_/5%N_2_, supplemented with 5 mM EGTA (Titriplex VI, Merck, Germany) and were incubated for 1h at 4 °C while gently shaking 90 times per minute. Pilot experiments showed that the concentration of BA in the slices gradually decreased during washing up to 2 h in Ca^2+^-free and Mg^2+^-free HBSS with EGTA, with a minimum after 1 h, suggesting that a part of the BA resided in bile canaliculi formed by tight hepatocyte junctions that are sensitive to Ca^2+^-depletion, which can be released after 1-h incubation in Ca^2+^ and Mg^2+^-free conditions. Therefore, a washing step of 1 h in Ca^2+^-free and Mg^2+^-free HBSS with EGTA was chosen as the most optimal. After this washing step, the slices were collected and stored in −80 °C until analysis. In those experimental groups where bile acids were not added to the medium, 3 slices were pooled together for the analysis, as the bile acid content of one slice was below the detection limit. In experimental groups with the addition of bile acids to the medium, individual slices were collected in separate 1.5-ml Eppendorf tubes. Collected PCLS were homogenized for 45 s in 450 μL of 70% methanol and centrifuged for 15 min at 15 °C and 13,000 rpm. The supernatant (350 μL) was collected to a new microcentrifuge tube and the pellet was used for further protein analysis. The TBA concentration in the sample was measured using Total Bile Acids Assay Kit (Diazyme, USA) according to the manufacturer’s instructions with slight modifications. The collected supernatant was evaporated at 35 °C using the CentriVap Concentrator (Labconco, USA) and the formed sample pellet containing bile acids was reconstituted in 70 μL of Milli-Q water and stored at −20 °C until analysis. After thawing, 5 μL of the sample was mixed with 135 μL of the Thio-NAD (>0.1 mM) buffer in a clear 96-well plate and incubated in the dark at 37 °C for 3 min. Subsequently, 45 μL of the 3-α-HSD (>2 kU/L), NADH (>0.1 mM) buffer was added to each well and the absorbance was read at 37 °C after 5 and 30 min at 405 nm. TBA concentration in the sample was calculated using the equation provided in the kit using conjugated cholic acid as a standard.

### Determination of individual bile acid concentrations by LC/MS

The concentration of DCA and CDCA, TDCA, GDCA, TCDCA, GCDCA. TCA and GCA was determined via LC-MS/MS in the same samples as used for the TBA assay and in appropriate calibration samples, using an Acquity UPLC system (Waters, Milford, MA, USA) coupled to a Xevo TQ-S (Waters) triple quadrupole mass spectrometer. The compounds were separated using a Zorbax Eclipse Plus C18 analytical column (Rapid Resolution HD 1.8 μm; 50 × 2.1 mm, Agilent, USA). The elution gradient was as follows: 0 min 30% B; 2–2.5 min 80% B; and 2.6–4 min 30% B. Solvent A consisted of 0.1% formic acid in water and Solvent B consisted of 0.1% formic acid in acetonitrile. The column temperature was set at 40 °C, and the flow rate was 500 µl/min. The effluent from the UPLC was passed directly into the electrospray ion source. Negative electrospray ionization was achieved using nitrogen as a desolvation gas with ionization voltage at 3.0 kVolt. The source temperature was set at 500 °C and argon was used as collision gas. Detection of the bile acids was based on isolation of the deprotonated molecular ion, [M–H]^−^ after which MS/MS fragmentations and multi reaction monitoring (MRM) were carried out. The following MRM transitions were used: for TDCA and TCDCA *m*/*z* 498.1(parent ion) to *m*/*z* 498.1 and 80.2 (both product ions), for GDCA *m*/*z* 448.3(parent ion) to *m*/*z* 448.3 and 74.0 (both product ions), for GCDCA *m*/*z* 448.3(parent ion) to *m*/*z* 386.8 and 74.0 (both product ions), for DCA *m*/*z* 391.2(parent ion) to *m*/*z* 391.2 and 345.0 (both product ions), for CDCA *m*/*z* 391.2(parent ion) to *m*/*z* 391.2 and 373.2 (both product ions), for TCA *m*/*z* 514.1(parent ion) to *m*/*z* 514.1 and 80.2 (both product ions), for GCA *m*/*z* 464.2(parent ion) to *m*/*z* 464.2 and 73.9 (both product ions).

### RNA isolation and cDNA synthesis

RNA isolation from the slices was performed using the Maxwell^®^ 16 LEV Total RNA purification kit (Promega, The Netherlands) with a Maxwell^®^ 16 LEV Instrument. After isolation, the RNA quality fulfilled the criteria of a 260/280 ratio of ~2 and 260/230 ratio of 2.0–2.2. The concentration was measured using the ND-100 spectrophotometer (Fisher Scientific, The Netherlands). TaqMan reverse Transcription Reagents Kits (Applied Biosystems, Foster City, CA) were used to generate cDNA from RNA. cDNA was generated in the Eppendorf master cycler (Hamburg, Germany) with the gradient at 20 °C for 10 min, 42 °C for 30 min, 20 °C for 12 min, 99 °C for 5 min and 20 °C for 5 min. RT-PCR was used to determine relative Bsep, Fxr and Ntcp mRNA expression levels. Primers used: *Bsep*, 5′-TGGAAAGGAATGGTGATGGG-3′(F), 3′-CAGAAGGCCAGTGCATAACAGA-5′(R); *Fxr*: 5′-CCAACCTGGGTTTCTACCC-3′(F), 3′-CACACAGCTCATCCCCTTT-5′(R); *Ntcp*; 5′-CTCCTCTACATGATTTTCCAGCTTG-3′(F), 3′-CGTCGACGTTCGTTCCTTTTCTTG-5′(R). RT-PCR was performed using SYBR Green with the ABI Prism 7900HT sequence detection system with 1 cycle of 10 minutes at 95 °C, 40 cycles of 15 s at 95 °C and 25 s at 60 °C, with a dissociation stage (15 s at 95, 60 and 95 °C). Sample Ct values were normalized using *Gapdh* as a housekeeping gene (5′-CGCTGGTGCTGAGTATGTCG-3′(F), 3′-CTGTGGTCATGAGCCCTTCC-5′(R)). The melt curve analysis was used to assess whether a single product was produced after RT-PCR. Data are expressed as a fold change compared to the non-treated control after 48 h of incubation.

### Statistics

A minimum of three different rat livers were used for each experiment, using slices in triplicates from each liver. Statistical testing was performed with two-way repeated measures ANOVA with individual rats as random effect. We performed a Tukey HSD post hoc test for pairwise comparisons. Correlation coefficients were calculated using Pearson’s R. A *p* value of ≤0.05 was considered to be significant. In all graphs the mean values and standard error of the mean (SEM) are shown.

## Results

### Viability of PCLS upon drug exposure

**Fig. 1:**
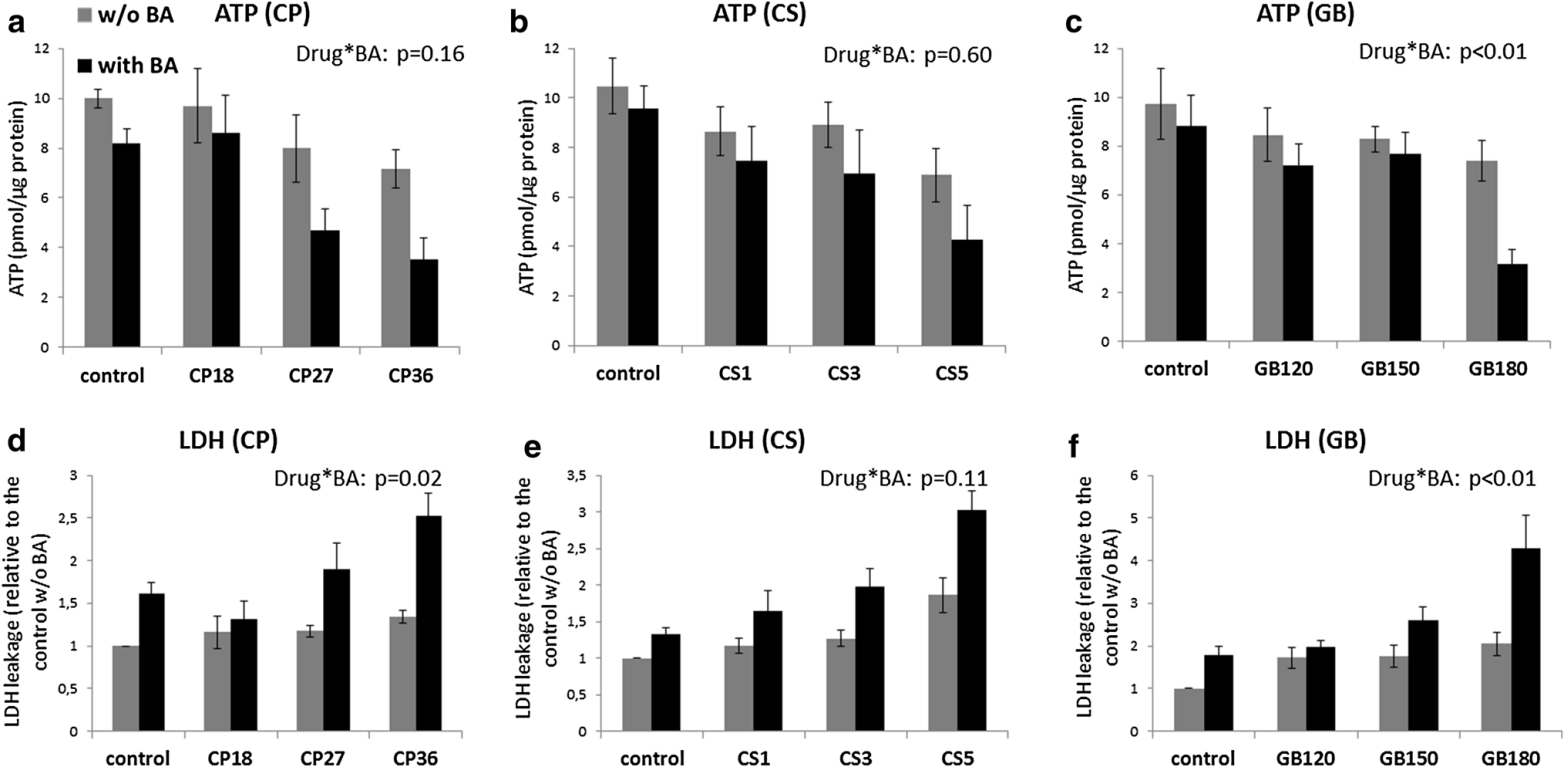
ATP content of PCLS after 48-h incubation (**a**–**c**) and LDH leakage from PCLS to incubation medium after 48-h incubation (**d**–**f**) with different drugs, in the absence (*gray bars*) or presence (*black bars*) of BA. ATP content is expressed in absolute values. LDH leakage is expressed as relative values to the control without BA. *Graphs* represent the mean values ± SEM of 4–5 experiments, using 3 PCLS for each group in every experiment. *p* values for interaction effects BA*drug are shown

The viability of PCLS following 48-h incubation with cholestatic drugs in the presence or absence of the BA mix was assessed by analysis of ATP content and LDH leakage to the incubation medium. The BA mix itself did not significantly decrease the ATP content of PCLS after 48 h (Fig. 1a–c). However, the LDH leakage increased slightly but significantly in the presence of BA (Fig. 1d–f). Addition of BA to the incubation medium increased the toxicity of the higher doses of CP, CS and GB (based on the decrease in ATP content and increase in LDH leakage); however, only in CP-treated groups (based on LDH) and GB-treated groups (based on ATP and LDH) it was found to be statistically significant. For other drug-treated groups a borderline significant trend was found.

### Bile acid accumulation in PCLS

**Fig. 2:**
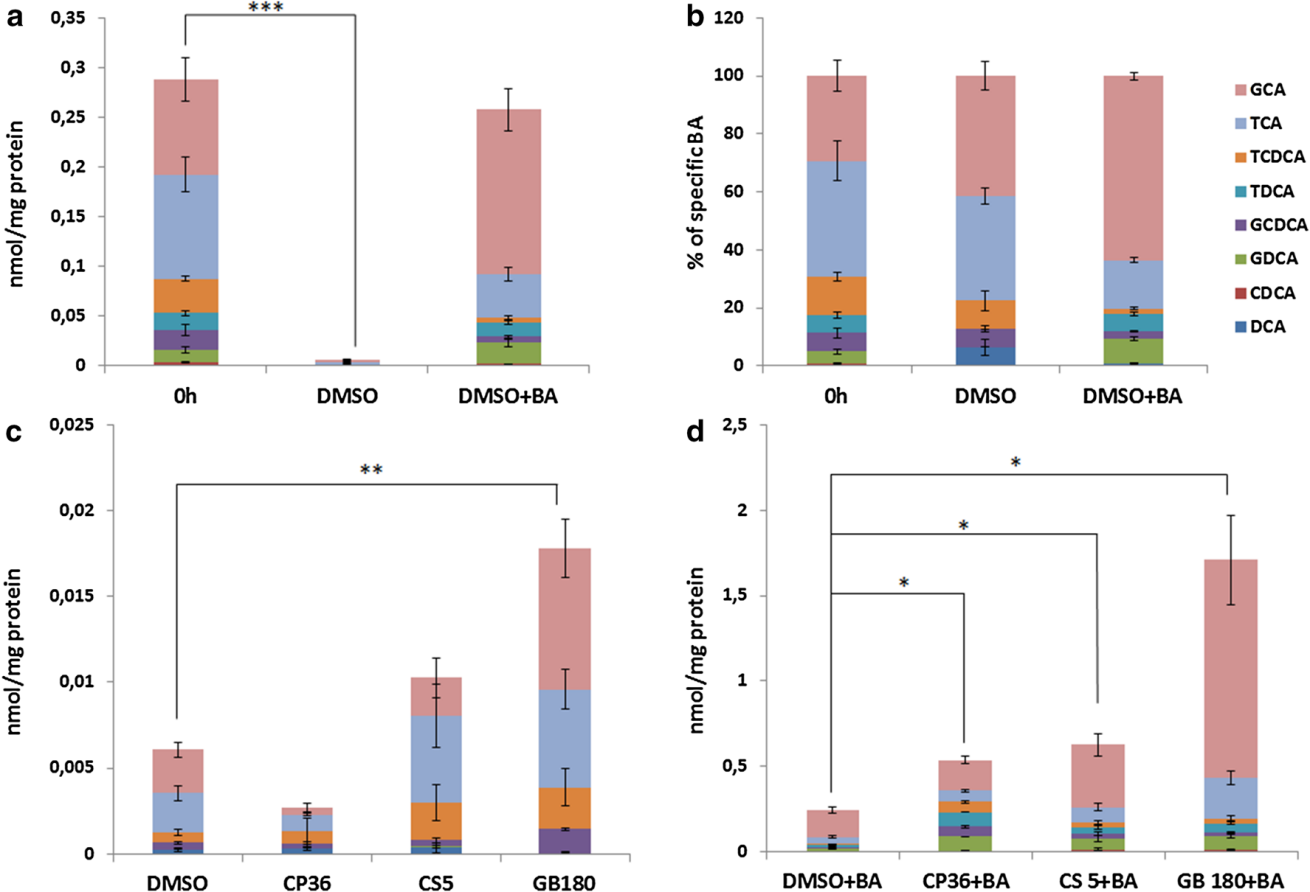
Concentration of eight different BA (GCA, TCA, TCDCA, TDCA, GCDCA, GDCA, CDCA, DCA) in fresh PCLS and in PCLS incubated for 48 h with or without BA, expressed in absolute values (**a**) or in percentage of total BA (**b**). Concentration of eight different BA in PCLS incubated for 48 h with the solvent DMSO or one of the cholestatic drugs CP36, CS5, GB180 in the absence (**c**) or presence (**d**) of BA. Data are expressed in absolute values. *Graphs* represent mean values ± SEM of 10–11 experiments for **a, b** and 3–5 experiments for **c, d**, using 3 PCLS for each group in every experiment

The BA content in PCLS incubated for 48 h without the addition of the BA mix, decreased by 82–98% compared to the fresh slice. The addition of the BA mix to the incubation medium, on the other hand, resulted in the maintenance of the physiological intracellular TBA concentration in PCLS after 48 h of incubation (Fig. 2a). The composition of the BA pool in PCLS incubated for 48 h with BA was only slightly different from that in fresh PCLS. For example, the concentration of GCA and GDCA was somewhat higher in slices incubated with the BA mix for 48 h compared to the fresh tissue, whereas the concentration of TCA and TDCA was lower in incubated PCLS compared to the non-incubated PCLS. In PCLS incubated without BA the total amount of these 8 BA in the slices was decreased by more than 90%, but the composition only changed to a minor extent (Fig. 2a, b).

**Fig. 3:**
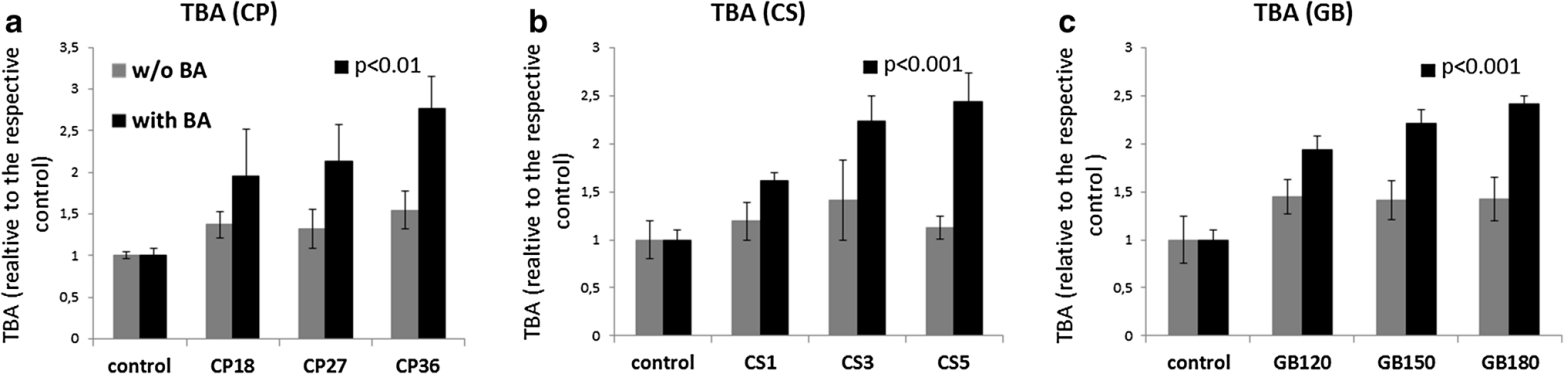
TBA content of PCLS after 48-h incubation with CP (**a**), CS (**b**) or GB (**c**), in the absence (*gray bars*) or presence (*black bars*) of BA. TBA content is expressed in relative values to the respective control (0.16 and 1.48 nmol/mg protein for the control without or with BA, respectively). Graphs represent the mean values ± SEM of 4–5 experiments, using 3 PCLS for each group in every experiment. *p* values for a dose-dependent increase in TBA content in BA + drug-treated groups are shown

The intracellular TBA concentration significantly increased in PCLS treated with CP, CS or GB in the presence of BA in a dose-dependent manner (Fig. 3). PCLS treated with the drug alone without BA, however, did not show a significant increase in TBA concentration, stressing the importance of the addition of the BA mix to the model. The concentrations of all eight BA measured individually increased after the treatment with the cholestatic drugs in the presence of BA (Table 2; Fig. 2d). The variation in the concentrations of individual BA between rats was high. However, the concentrations of every BA increased within every rat upon the drug treatment. The total concentration of the eight tested BA as measured by LC/MS in PCLS upon drug + BA-treatment was lower than the TBA concentration measured by the enzymatic assay, which can be due to the fact that some BA, such as muricholic acid, as well as sulfate and glucuronide conjugates of BA were not measured by LC/MS.

**Table 2:** Concentration and absolute amount of eight different BA in PCLS incubated for 48 h with the solvent DMSO or one of the cholestatic drugs CP36, CS5, GB180 ATP in the presence of BA

pmol/mg protein	DMSO + BA	CP36 + BA	CS 5 + BA	GB 180 + BA
DCA	1.4 ± 1.3 (0.8%)	3.9 ± 2.8 (0.6%)	7.5 ± 8.3 (1.3%)	5.7 ± 8.3 (1%)
CDCA	0.3 ± 0.7 (0.1%)	4.0 ± 5.1 (0.5%)	8.1 ± 10.8 (1.1%)	6.7 ± 11.0 (1.2%)
GDCA	20.2 ± 11.8 (8.5%)	80.8 ± 4.7 (15.5%)	61.1 ± 38.3 (9.4%)	82.0 ± 23.0 (6.7%)
GCDCA	6.6 ± 3.8 (2.9%)	60.7 ± 13.4 (10.7%)	28.4 ± 24.9 (4.7%)	16.2 ± 9.0 (1.8%)
TDCA	13.5 ± 7.0 (6.0%)	83.8 ± 1.5 (16.3%)	38.6 ± 23 (6.2%)	56.2 ± 15.6 (5.1%)
TCDCA	4.9 ± 3.3 (2.2%)	60.2 ± 14.2 (10.4%)	29.0 ± 28.9 (4.3%)	30.3 ± 28.3 (4.2%)
TCA	41.4 ± 23.1 (16.5%)	64.5 ± 16.0 (12.1%)	90.2 ± 42.7 (14.4%)	235.8 ± 69.2 (13.8%)
GCA	156.5 ± 73.9 (63.0%)	180.3 ± 45.4 (33.8%)	364.3 ± 149.5 (58.6%)	1275.6 ± 454.7 (66.2%)
Total	245.0 ± 118.3 (100%)	538.1 ± 93.4 (100%)	627.2 ± 260.5 (100%)	1708.5 ± 448.1 (100%)

Data are expressed in absolute values. Table represents mean values ± SD (% of total) of 10–11 experiments for DMSO group and 3–5 experiments for drug-treated groups, using 3 PCLS for each group in every experiment

When incubated in the presence of BA, the relative abundance of the eight tested bile acids was similar in the CS5 + BA- and GB180 + BA-treated groups in comparison to the control, with the exception of CDCA, which increased 9 times upon drug exposure (Fig. 2d). The relative abundance of TCDCA and GCDCA was elevated upon CP36 + BA treatment (from 5 to about 20%), whereas that of TCA and GCA decreased (from 80% to about 45%) compared to the control. Furthermore, the relative abundance of CDCA increased four-fold, and of GDCA and TDCA increased 1.8- and 2.7-fold, respectively, in PCLS treated with CP36 + BA compared to the control (Table 2).

Exposure of PCLS to CS5 or GB180 in the absence of BA resulted in a small increase in the cumulative concentration of the tested BA (Fig. 2c). Surprisingly, the cumulative concentration of the eight tested BA in PCLS after CP36-treatment was lower compared to the non-treated control (Fig. 2c). However, this phenomenon was not shown with the measurement of TBA concentration, where the concentration of TBA in PCLS was slightly higher in CP36-treated group compared to the control. No major changes occurred in the relative abundance of the different BA upon PCLS exposure to CP36, CS5 or GB180 alone.

### Gene expression

**Fig. 4:**
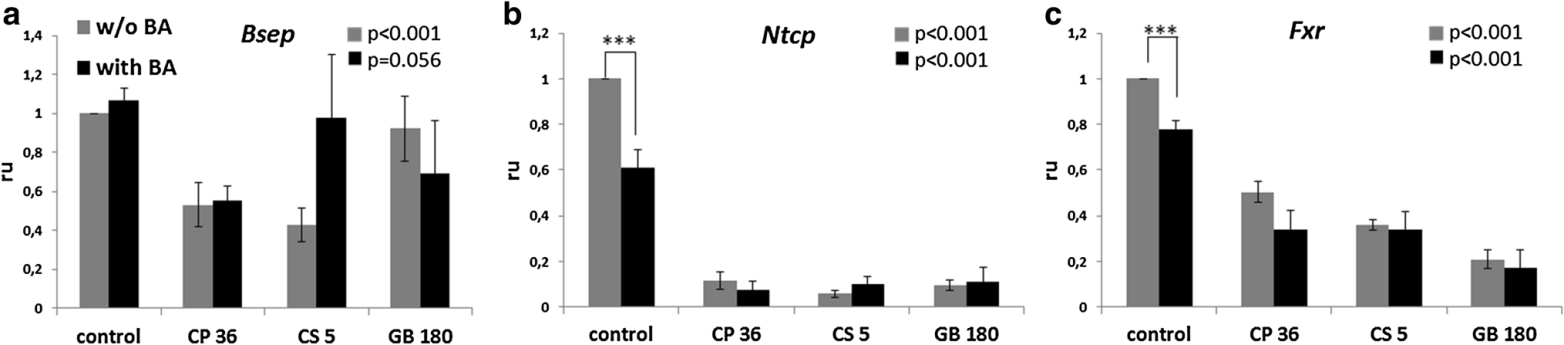
Gene expression of *Bsep* (**a**), *Ntcp* (**b**) and *Fxr* (**c**) normalized to the housekeeping gene *Gapdh*. Data expressed as relative values compared to the 48-h control without BA. *Graphs* represent mean values ± SEM of 3–4 experiments, using 3 PCLS for each group in every experiment. *p* values for a comparison between control and all drug-treated groups in the absence and presence of BA are shown

Changes in the expression of genes known to be involved in cholestasis, such as *Bsep, Ntcp* and *Fxr* have been investigated. NTCP is responsible for the basolateral uptake of bile acids, whereas BSEP is responsible for their efflux to the bile canaliculi on the apical side of hepatocytes. BA significantly decreased *Ntcp* gene expression to 60% in PCLS after 48 h of incubation. This effect of BA on *Ntcp* expression is known and has been described before. *Ntcp* gene expression in PCLS was significantly and strongly decreased to ca. 10% of the value of the controls after incubation with CP36, CS5 and GB180 treatment alone and was not further decreased in the presence of BA (Fig. 4b). *Bsep* gene expression showed some variation between and within experimental groups, and was significantly decreased in the drug alone-treated groups when all experiments were taken altogether, but not in the group drug + BA (Fig. 4a).

*Fxr* is thought to be a master regulator of BA homeostasis, acting on BA synthesis, conjugation and transport. In our study, *Fxr* expression was significantly downregulated by 20% in PCLS incubated with the BA mix for 48 h, with a further stronger decrease in PCLS treated with CP, CS or GB (Fig. 4c), with no difference between with and without BA.

### Correlation analysis

**Fig. 5:**
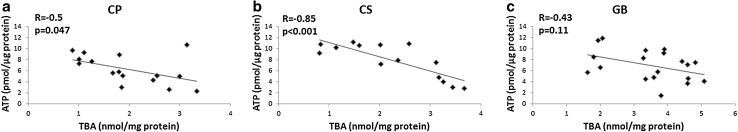
Correlation between ATP and TBA content of PCLS treated with CP (**a**), CS (**b**) or GB (**c**) in combination with BA. *Graphs* represent mean values ± SEM of 4–5 experiments, using 3 PCLS for each group in every experiment. *p* values and *R* values are shown

The decrease in viability (based on ATP content) of PCLS upon the exposure to CP and CS in combination with BA correlated with an increase in intracellular levels of BA, whereas no significant correlation was observed in the GB-treated groups (Fig. 5). The same results were obtained from the correlation analysis between LDH leakage and TBA content.

## Discussion

In this study rat PCLS were verified as a model to study drug-induced cholestasis (DIC) and pathological processes related to DIC using several biomarkers. Three drugs known to induce cholestasis were used as model compounds: chlorpromazine, cyclosporine A and glibenclamide (Stieger 2010) in the presence of a physiological bile acid mix. Earlier PCLS were used as a model to study early transcriptional changes associated with cholestasis using mouse and human liver tissue showing gene expression profiles related to pathological changes associated with cholestasis (Szalowska et al. 2013; Vatakuti et al. 2016). Here we suggest for the first time that these drugs cause cholestatic effects as they are more toxic in the presence of BA in rat PCLS and induce changes in the intracellular bile acid concentration and composition.

The presence of BA in their physiological concentrations and ratios may increase the validity of an in vitro model for DIC studies. For example, incubation of mouse PCLS with cholestatic drugs alone did not result in the elevation in intracellular BA concentration (Szalowska et al. 2013). Moreover, in our study we showed that without the addition of BA, PCLS excreted most of them during incubation, whereas the incubation of PCLS with the BA mix resulted in the maintenance of the physiological concentrations of BA in PCLS after 48 h of incubation. The observed shift to glycine conjugates after the incubation with the BA mix might be due to the small difference between the composition of the BA mix (which was based on the portal vein BA concentrations) and tissue values, as well as the more active production of glycine conjugates of BA during incubation compared to the production of tauroconjugates. This higher production of glycine conjugates might be explained by the higher substrate availability of glycine compared to the taurine precursor (L-cysteine) in the medium (http://www.thermofisher.com/) (Penttilä 1990). In addition, a shift between taurine and glycine conjugation was also found before in rats fed diet with different supplements (Park et al. 1999).

The secondary bile acids, such as DCA and LCA, are considered to be the most toxic BA due to their lipophilicity and, therefore, are important in the progression of bile acid-induced hepatic injury (Li and Chiang 2011; Schadt et al. 2016; Yang et al. 2013). However, they are not produced in the liver but formed in the intestinal track by anaerobic and/or aerobic bacteria (Alnouti 2009). Since in vitro liver models usually do not include the liver–intestine interaction part, the addition of these secondary BA to the BA mix is necessary to study the effects of these toxic BA and the shifts in ratios of certain BA during cholestasis.

High concentrations of CP, CS and GB in combination with the BA mix decreased the viability of PCLS, which correlated for CP and CS with the increase in the intracellular concentrations of BA. The increase in the total concentration of BA upon the exposure to cholestatic drugs as well as changes in the fractions of the eight most prevalent BA in the body has been analyzed. We showed that all three cholestatic drugs CP, CS and GB caused a dose-dependent increase in BA in PCLS, but this resulted in different profiles of accumulated BA. For example, CP-treatment increased the relative abundance of CDCA and its conjugates GCDCA and TCDCA, as well as DCA conjugates GDCA and TDCA, whereas the abundance of GCA and TCA was decreased. CS and GB, on the other hand, did not significantly change the composition of the BA pool with respect to CA and CDCA conjugates, but the relative abundance of CDCA itself and that of the secondary BA DCA were increased. DCA and CDCA are known to be more toxic than the other measured BA due to their lipophilicity and, therefore, the increase in their intracellular concentration in PCLS treated with cholestatic drugs, might at least partly be responsible for the toxicity observed. The differences in the composition of the intracellular BA pool following the exposure to different cholestatic drugs may be explained by the different abilities of the individual drugs to inhibit the BSEP-mediated transport of certain BA (Yang et al. 2013).

In this study, we did not find a threshold value of the intracellular concentration of TBA above which the viability of PCLS decreases. Different drugs decreased PCLS viability at different intracellular levels of BA, showing some variability also within their own group. These data might indicate that the toxicity depends not on the elevation in the total BA, but more likely is attributed to the increase in the concentration and relative abundance of certain toxic BA, such as LCA, DCA and its conjugates and CDCA. We have shown the increase in the relative abundance of DCA, DCA conjugates and CDCA upon the treatment with toxic concentrations of cholestatic drugs. However, to show the correlation between the viability of PCLS and the increase in the concentration of these BA (with the possibility to find a threshold value), further analysis with various concentrations of drugs needs to be performed.

Previous studies on sandwich-cultured hepatocytes (SCH) demonstrated higher toxicity of CS and GB in the presence than in the absence of BA. In our study, CS concentrations that led to the decrease in viability and increase in intracellular BA concentrations were two- to threefolds lower (Chatterjee et al. 2014b; Ogimura et al. 2011; Oorts et al. 2016) than that found with rat or human SCH. In PCLS, IC_50_ values of CP and GB in the presence of BA were similar to (for CP) or 1.5–3-folds higher (for GB) than those in rat SCH (Chatterjee et al. 2014b; Ogimura et al. 2011). Nevertheless, none of these studies measured BA accumulation in hepatocytes to confirm BA-dependent hepatotoxicity. Even though Ansede et al. showed the possibility to study intracellular accumulation of BA upon CS and GB treatment in SCH, further studies using other toxicity parameters to test BA-dependent toxicity have not been performed (Ansede et al. 2010). Future studies comparing PCLS to other in vitro models and using PCLS model to test drugs that were not recognized as cholestatic by these other models would be of interest.

Elevated BA levels are considered to be one of the key triggers in DILI development. However, in many cases, altered transporter functions coincide with other mechanisms of cytotoxicity and liver injury. The ability of BA to trigger cytotoxic effects has been shown in various studies (Attili et al. 1986; Palmeira and Rolo 2004; Rolo et al. 2000; Woolbright et al. 2015). For example, certain BA was shown to impair mitochondrial function by altering the transmembrane potential and mitochondrial permeability transition (Rolo et al. 2000). Moreover, a recent study on primary human hepatocytes (PHH) showed that exposure of PHH to high concentration of GCDCA leads to necrosis. However, the BA concentrations used in this study far exceeded the serum concentrations measured in cholestatic patients (Woolbright et al. 2015). Serum BA values are not always representative for the intracellular BA concentrations, since they represent the net effect of the uptake of BA into hepatocytes, which can be also inhibited without cholestasis, and the basolateral efflux of BA due to high intracellular concentrations during cholestasis.

In our study, low non-toxic doses of cholestatic drugs moderately increased the intracellular BA concentration in PCLS, possibly indicating inhibition of the excretion of BA by BSEP. However, high doses of drugs are likely to provoke various other toxicological mechanisms aside BSEP inhibition that contribute to the overall toxicity observed. For example, high doses of CP, CS and GB without the presence of BA in the medium slightly decreased ATP levels and increased LDH leakage in PCLS, which cannot be explained by the small increase in intracellular BA levels as they are still far below the physiological levels, represented by the *t* = 0 samples and the values at 48-h incubation with BA (Figs. 2c, d, 3). Therefore, while there are indications that BSEP inhibition and as a consequence BA accumulation are important players in DILI initiation, the progression of liver injury likely also involves other key adverse events that lead to the disturbance of the normal cell physiology.

*Fxr* is a member of the nuclear receptor subfamily and is directly involved in BA homeostasis (Claudel et al. 2005; Modica et al. 2010; Schadt et al. 2016). It was reported that *Fxr* regulates both bile acid synthesis and transport. For example, activation of *Fxr* by BA leads to the downregulation of *Cyp7a1* and *Cyp8b1* involved in BA synthesis (Li and Chiang 2011; Schadt et al. 2016). Moreover, activation of *Fxr* by an intracellular increase in BA concentration suppresses the hepatic influx transporters NTCP and OATP and upregulates the major bile acid efflux transporter BSEP. This downstream cascade of responses is playing a protective role directed on the reduction of intracellular BA concentration and, as a result, prevention of the bile acid-induced cytotoxicity.

Incubation of PCLS with the BA mix resulted in the slight downregulation of *Fxr* gene. This may be due to the increased levels of GDCA as it was shown that GDCA decreased the expression of *Fxr* in human cholangiocarcinoma cells in vitro (Dai et al. 2011). Moreover, induced expression of human and mouse *Fxr* by *Fxr* agonists GW4064 and CDCA was reversed by LCA or GDCA (Dai et al. 2011; Yu et al. 2002). The secondary BA, DCA and LCA have a higher affinity to the *Fxr* receptor than, for example CDCA, that increases the expression of *Fxr* and activates it (Dai et al. 2011; Paumgartner et al. 2004; Yu et al. 2002). In our study, the concentration of DCA and its conjugates in PCLS incubated with the BA mix was more than 100-fold higher than in the control slices without the addition of BA that might explain the difference in *Fxr* expression and activation between the two groups. This also indicates that the regulation of *Fxr* expression and its target genes depends on a defined balanced of agonists and antagonists in the milieu.

Exposure of PCLS to CP, CS or GB resulted in a very strong downregulation of *Fxr*, which is in line with previous studies on mouse and human PCLS that demonstrated FXR downregulation by cholestatic compounds (Szalowska et al. 2013; Vatakuti et al. 2016). Regulation of *Fxr* in hepatocytes and enterocytes is a complex process of a multifactorial nature that can be also influenced by other factors not directly involved in bile acid homeostasis. For example, a given drug can directly disrupt *Fxr* function or can influence its expression indirectly by triggering inflammatory and cytotoxic processes in the liver (Rodrigues et al. 2014; Szalowska et al. 2013). Various cholestatic drugs, including CP and CS, have been shown to cause ER stress, oxidative stress, apoptosis, trigger inflammatory responses and influence β-oxidation in the liver (Anthérieu et al. 2013; Szalowska et al. 2015; Vatakuti et al. 2016). These pathological processes, in turn, can lead to the downregulation of *Fxr*, as well as other nuclear receptors (Szalowska et al. 2013). Moreover, the concentration of secondary BA DCA and its conjugates increased 3 to five-folds in PCLS treated with cholestatic drugs that might have further contributed to *Fxr* downregulation.

The *Ntcp* expression in rat PCLS treated with the BA mix was 40% lower compared to the non-treated control, which is in line with previously reported data, where it was shown that BA negatively regulates *Ntcp* expression. The effects of BA on *Ntcp* are thought to be mediated via *Fxr* stimulation and, as a result, SHP pathway activation (Modica et al. 2010; Schadt et al. 2016). Furthermore, BA can increase SHP stability by inhibiting its proteasomal degradation in an extracellular signal-regulated kinase (ERK)-dependent manner (Miao et al. 2009). In our study *Ntcp* downregulation by the BA mix could be of *Fxr*-dependent or -independent manner, since *Fxr* expression was also downregulated in the BA-treated group compared to the non-treated control. The *Ntcp* expression was shown to be regulated also via other signaling pathways involving transcription factors, such as HNF4α, HNF3β, HNF1α or the glucocorticoid receptor (Kosters and Karpen 2008; Matsubara et al. 2012). Moreover, *Ntcp* expression in the non-treated control, on the other hand, could have been upregulated due to the low concentrations of BA present in the system. All tested cholestatic drugs induced a downregulation of *Ntcp* expression, which can be considered as an adaptive response to the increase in intracellular concentration of BA. Moreover, the three drugs themselves were found to downregulate *Ntcp* in the absence of BA.

Inhibition of BSEP is thought to play a crucial role in drug-induced cholestasis (Vinken et al. 2013; Yang et al. 2013). Cholestatic drugs can directly inhibit BSEP activity by binding to the transporter protein in a competitive manner (Kosters and Karpen 2008; Pauli-Magnus and Meier 2006; Stieger 2010). Some previous studies have shown that cholestatic drugs may also reduce the expression of *Bsep* mRNA in mouse and human PCLS (Szalowska et al. 2013; Vatakuti et al. 2016), whereas another study on rats did not reveal any changes in *Bsep* expression after CS and sirolimus treatment (Bramow et al. 2001). In our study *Bsep* expression was reduced in slices treated with CP (with or without BA mix) or CS-alone. GB treatment, however, did not result in any significant changes in *Bsep* mRNA levels. Moreover, it is thought that genetic polymorphism in the expression of BSEP or mutations in this transporter increase the risk of the development of DIC and is associated with idiosyncratic cholestatic episodes in some of the patients taking a potentially cholestatic drug (Chalasani and Björnsson 2010).

In summary, we showed that rat PCLS exposed to cholestatic drugs in the presence of a physiological BA mix reflect various changes associated with cholestasis, such as decrease in hepatocyte viability, accumulation of BA and changes in the expression of genes known to play a role in cholestasis. Therefore, we believe that PCLS represent a physiological and valuable model to identify potential cholestatic compounds and elucidate mechanisms underlying DIC and DILI. The toxicity of cholestatic drugs was correlated with the accumulation of BA, and especially DCA and CDCA and their conjugates, but to a different extent for the different drugs, indicating that BA toxicity is not the only cause for the toxicity of cholestatic drugs. This study was performed with rat PCLS and, therefore, needs to be further verified with human PCLS, to avoid species-specific differences with respect to the cholestatic action of the tested compounds. Moreover, our study supports the use of a combination of several biomarkers to identify the cholestatic potential of a drug, since every one of them apart might not be sufficiently predictive or specific.

## References

[CR1] Alnouti Y (2009). Bile acid sulfation: a pathway of bile acid elimination and detoxification. Toxicol Sci.

[CR2] Ansede JH, Smith WR, Perry CH, Claire RL St, Brouwer KR (2010). An in vitro assay to assess transporter-based cholestatic hepatotoxicity using sandwich-cultured rat hepatocytes. Drug Metab Dispos.

[CR3] Anthérieu S, Azzi PB, Dumont J, Abdel-Razzak Z, Guguen-Guillouzo C, Fromenty B, Robin M, Guillouzo A (2013). Oxidative stress plays a major role in chlorpromazine-induced cholestasis in human HepaRG cells. Hepatology.

[CR4] Attili AF, Angelico M, Cantafora A, Alvaro D, Capocaccia L (1986). Bile acid-induced liver toxicity: Relation to the hydrophobic-hydrophilic balance of bile acids. Med Hypotheses.

[CR5] Bramow S, Ott P, Thomsen Nielsen F, Bangert K, Tygstrup N, Dalhoff K (2001). Cholestasis and regulation of genes related to drug metabolism and biliary transport in rat liver following treatment with cyclosporine A and sirolimus (Rapamycin). Pharmacol Toxicol.

[CR6] Chalasani N, Björnsson E (2010). Risk factors for idiosyncratic drug-induced liver injury. Gastroenterology.

[CR7] Chatterjee S, Bijsmans IT, van Mil SW, Augustijns P, Annaert P (2014). Toxicity and intracellular accumulation of bile acids in sandwich-cultured rat hepatocytes: role of glycine conjugates. Toxicol in Vitro.

[CR8] Chatterjee S, Richert L, Augustijns P, Annaert P (2014). Hepatocyte-based in vitro model for assessment of drug-induced cholestasis. Toxicol Appl Pharmacol.

[CR9] Claudel T, Staels B, Kuipers F (2005). The farnesoid X receptor: a molecular link between bile acid and lipid and glucose metabolism. Arterioscler Thromb Vasc Biol.

[CR10] Dai J, Wang H, Shi Y, Dong Y, Zhang Y, Wang J (2011). Impact of bile acids on the growth of human cholangiocarcinoma via FXR. Journal of Hematology Oncology.

[CR11] de Graaf I AM, Olinga P, de Jager M H, Merema MT, de Kanter R, van de Kerkhof EG, Groothuis GMM (2010). Preparation and incubation of precision-cut liver and intestinal slices for application in drug metabolism and toxicity studies. Nat Protocols.

[CR12] Kosters A, Karpen SJ (2008). Bile acid transporters in health and disease. Xenobiotica.

[CR13] Li T, Chiang JYL (2011). Bile acid signaling in liver metabolism and diseases. Journal of Lipids.

[CR14] Matsubara T, Li F, Gonzalez FJ (2012) FXR signaling in the enterohepatic system. Mol Cell Endocrinol 368:17–29 10.1016/j.mce.2012.05.004 PMC3491147 22609541

[CR15] Miao J, Xiao Z, Kanamaluru D, Min G, Yau PM, Veenstra TD, Ellis E, Strom S, Suino-Powell K, Xu HE, Kemper JK (2009). Bile acid signaling pathways increase stability of Small Heterodimer Partner (SHP) by inhibiting ubiquitin–proteasomal degradation. Genes Dev.

[CR16] Modica S, Gadaleta RM, Moschetta A (2010). Deciphering the nuclear bile acid receptor FXR paradigm. Nucl Recept Signal.

[CR17] Ogimura E, Sekine S, Horie T (2011). Bile salt export pump inhibitors are associated with bile acid-dependent drug-induced toxicity in sandwich-cultured hepatocytes. Biochem Biophys Res Commun.

[CR18] Oorts M, Baze A, Bachellier P, Heyd B, Zacharias T, Annaert P, Richert L (2016). Drug-induced cholestasis risk assessment in sandwich-cultured human hepatocytes. Toxicol in Vitro.

[CR19] Padda MS, Sanchez M, Akhtar AJ, Boyer JL (2011). Drug-induced cholestasis. Hepatology.

[CR20] Palmeira CM, Rolo AP (2004). Mitochondrially-mediated toxicity of bile acids. Toxicology.

[CR21] Park T, Oh J, Lee K (1999). Dietary taurine or glycine supplementation reduces plasma and liver cholesterol and triglyceride concentrations in rats fed a cholesterol-free diet. Nutr Res.

[CR22] Pauli-Magnus C, Meier PJ (2006). Hepatobiliary transporters and drug-induced cholestasis. Hepatology.

[CR23] Paumgartner G, Keppler D, Leuschner U, Stiehl A (eds) (2004) Bile acid biology and its therapeutic implications. Springer

[CR24] Penttilä KE (1990). Role of cysteine and taurine in regulating glutathione synthesis by periportal and perivenous hepatocytes. Biochem J.

[CR25] Qiu X, Zhang Y, Liu T, Shen H, Xiao Y, Bourner MJ, Pratt JR, Thompson DC, Marathe P, Humphreys WG, Lai Y (2016). Disruption of BSEP function in HepaRG Cells Alters Bile acid disposition and is a susceptive factor to drug-induced cholestatic injury. Mol Pharmaceutics.

[CR26] Rodrigues AD, Lai Y, Cvijic ME, Elkin LL, Zvyaga T, Soars MG (2014). Drug-induced perturbations of the bile acid pool, cholestasis, and hepatotoxicity: mechanistic considerations beyond the direct inhibition of the bile salt export pump. Drug Metab Dispos.

[CR27] Rolo AP, Oliveira PJ, Moreno AJM, Palmeira CM (2000). Bile Acids Affect Liver Mitochondrial Bioenergetics: Possible Relevance for Cholestasis Therapy. Toxicol Sci.

[CR28] Schadt HS, Wolf A, Pognan F, Chibout S, Merz M, Kullak-Ublick GA (2016). Bile acids in drug induced liver injury: Key players and surrogate markers. Clinics Research in Hepatology Gastroenterology.

[CR29] Starokozhko V, Abza GB, Maessen HC, Merema MT, Kuper F, Groothuis GM (2015). Viability, function and morphological integrity of precision-cut liver slices during prolonged incubation: Effects of culture medium. Toxicol in Vitro.

[CR30] Stieger B (2010). Role of the bile salt export pump, BSEP, in acquired forms of cholestasis. Drug Metab Rev.

[CR31] Szalowska E, Stoopen G, Groot MJ, Hendriksen PJM, Peijnenburg AACM (2013). Treatment of mouse liver slices with cholestatic hepatotoxicants results in down-regulation of Fxr and its target genes. BMC Med Genomics.

[CR32] Szalowska E, Pronk TE, Peijnenburg AACM (2015). Cyclosporin A induced toxicity in mouse liver slices is only slightly aggravated by Fxr-deficiency and co-occurs with upregulation of pro-inflammatory genes and downregulation of genes involved in mitochondrial functions. BMC Genomics.

[CR33] Vatakuti S, Olinga P, Pennings JLA, Groothuis GMM (2016) Validation of precision-cut liver slices to study drug-induced cholestasis: a transcriptomics approach. Arch Toxicol:1–12 10.1007/s00204-016-1778-8 PMC5316400 27344345

[CR34] Vickers AEM, Fisher RL (2013). Evaluation of drug-induced injury and human response in precision-cut tissue slices. Xenobiotica.

[CR35] Vinken M, Landesmann B, Goumenou M, Vinken S, Shah I, Jaeschke H, Willett C, Whelan M, Rogiers V (2013). Development of an adverse outcome pathway from drug-mediated bile salt export pump inhibition to cholestatic liver injury. Toxicological sciences : an official journal of the Society of Toxicology.

[CR36] Woolbright BL, Dorko K, Antoine DJ, Clarke JI, Gholami P, Li F, Kumer SC, Schmitt TM, Forster J, Fan F, Jenkins RE, Park BK, Hagenbuch B, Olyaee M, Jaeschke H (2015). Bile acid-induced necrosis in primary human hepatocytes and in patients with obstructive cholestasis. Toxicol Appl Pharmacol.

[CR37] Yang K, Köck K, Sedykh A, Tropsha A, Brouwer KLR (2013). An updated review on drug-induced cholestasis: Mechanisms and investigation of physicochemical properties and pharmacokinetic parameters. J Pharm Sci.

[CR38] Yu J, Lo J, Huang L, Zhao A, Metzger E, Adams A, Meinke PT, Wright SD, Cui J (2002). Lithocholic Acid Decreases Expression of Bile Salt Export Pump through Farnesoid X Receptor Antagonist Activity. J Biol Chem.

